# Address at the Commencement of the Ohio College of Dental Surgery

**Published:** 1869-02

**Authors:** G. W. Keely


### DENTAL REGISTER.

Vol. XXIII.] FEBRUARY, 1869. [No. 2.

### Original Communications.

### ADDRESS AT THE COMMENCEMENT OF THE OHIO COLLEGE OF DENTAL SURGERY.

BY DR. G. W. KEELY,

President of the Board of Trustees, March 3, 1869.

Young Gentlemen: With the close of this evening ends the twenty-third annual session of the Ohio College of Dental Surgery.

For the past five months you have devoted your time and energy in completing the regular course of studies prescribed by the officers of this institution; and the Faculty now recommend you as worthy recipients of the highest honors she can bestow.

Your perseverance and industry has enabled you to conquer every obstruction that appeared in your way, and this fact should encourage you to go on in the good work; never faltering until you have attained an honorable position in your profession.

And now that you have assumed a new position, it should be your aim to excel in every department of Dental science; not being content in occupying a medium standing among your professional brethren, but by an honorable and gentlemanly manner in performing every duty pertaining to your calling as a professional man, and your devotion to your specialty, be able to add something new and valuable to the common stock of knowledge, which will benefit your profession and prove a blessing to mankind.

In addressing you as gentlemen, we do not wish you to imagine we use the title in the empty perverted sense it is so often used in the ordinary intercourse in life. In this free land of ours, where titles and distinctions of rank have no existence, the term is applied to men of education and good breeding, without reference to occupation.

An officer in the army of the United States may be tried by his brother officers for conduct unbecoming a gentleman, and if found guilty, he is dismissed the service in disgrace. If any man is charged with not being a gentleman, it is considered the most degrading that can be preferred against him.

In our "Dental Associations" every member subscribes to a code of Ethics, for the violation of which he is liable to be tried for conduct unbecoming a professional gentleman, and if found guilty he is expelled.

We do not desire to discuss this subject at length, but more particularly consider it in its highest acceptation.

A gentleman in the true sense is uniformly known and distinguished as the soul of honor, generous to a fault, with refined feelings and dignified deportment on all occasions; one in whose nature no mean and selfish feelings find lodgment; one whose self respect makes him careful not to offend those with whom he comes in contact.

A gentleman is possessed with true manly courage, moral and physical, and dares to do right.

Whether you are engaged in the duties of your profession, or enjoying the pleasures of social life, always be gentlemen.

To be gentlemen, you must be true men, nothing more nothing less.

The real gentleman must be honest, earnest and kind; honest in avoiding all pretence, earnest in pursuit of duty, kind in extending charity to all. Such a man is as far removed from the sycophant as from the boor, and he who is thus honestly in earnest to please, can not fail to exhibit true gentlemanly kindness.

But, my young friends, do not for one moment ever imagine you can permanently promote your pecuniary or worldly interest as a member of an honorable profession by the least infraction of those strict rules of gentlemanly conduct.

The profession to which you are welcomed to-night, has for its object the cultivation and practice of the healing art—your mission is to *save*, not *destroy* those invaluable organs God has given his creatures for a wise and benificent purpose.

No other profession has advanced more rapidly within the last quarter of a century than the one you are just entering.

Good honest men are laboring with a zeal almost superhuman to clear away the rubbish and expel the many errors that exist, that the *pure gold* may be exposed to the sun-light. Truly the advancement of science in the Dental profession has been almost miraculous, and it is your mission, young gentlemen, to push on the car of progress until we reach that degree of perfection so " devoutly to be wished for."

As you go from these Halls, for a time you are like soldiers called from the training school to the realities of the battle-field. Like them you are armed for your warfare, and have acquired a practical as well as a theoretical knowledge of your profession. But this alone is not sufficient to insure success, and build up an honorable reputation. Energy and perseverance are the golden keys that unlock the volume of History, reveals the riches of the past, and unfolds the secrets of the future. Like the fig-tree, they bear fruit all the year round, but he who would partake of the fruit must cultivate the tree.

The simple mention of perseverance pre-supposes opposition. This is the case in every profession and calling, as well as ours; indeed, life is made up of responsibility, failure and success. Every rose has its thorn, every pleasure (save that of doing good) its pain; but he who has not witnessed the darkness can not half enjoy the light, and he who has not known opposition can not realize success.

We would impress upon your minds that perseverance is a necessary virtue, not only that we may gain fresh laurels, but that we may retain those already won. If you do not practice the virtue of perseverance, the evil of indolence will soon control all the springs of action, and sad indeed will be the wreck of manhood's noblest aspirations.

Chesterfield tritely said, "Indolence is a sort of suicide;" and most assuredly inactivity is to-day the greatest enemy of man, the adversary of progress, the guardian of ignorance, the fruitful parent of vice and poverty. Study then to show yourselves "workmen that need not be ashamed," and ever bear in mind that in every profession, as Webster once said, "There is room enough at the top." You can profit by the experience of those who have labored in the past, and by the virtue of energy and perseverance, find room as Webster did, at the top.

To insure success, you must devotedly love your profession, and take an active interest in all that pertains to it; for ours is a noble profession, a beautiful art. To relieve suffering, to arrest disease or remove its cause, to remedy the defects of nature, to imitate her noblest handi-work, to rob Time of his premature spoils, to embalm the smiles of youth and beauty, and perpetuate them to old age; all these pertain to our calling, and constitute just grounds why we should love our profession.

We would particularly warn you not to be in too great a hurry to get rich, for at best it is a slow process in our calling.

In this fast age, when everything seems inclined to move with telegraphic speed, when men accumulate princely fortunes in a day, the forests are cleared as with a whirlwind; railroads are stretching their iron arms far toward the Pacific, opening the way for teeming millions to cultivate the rich soil, and dig deep into the bowels of the earth to extract its treasures; towns and cities rising as if by magic, inviting the fearless to accumulate riches; many young men are influenced to depart from the path of honor and virtue, in the effort to outstrip their fellows in the race for wealth.

Many otherwise truly good men have split upon this rock; finding themselves favored with an extensive practice, and surrounded by competitors, they hurry through their operations, having only a selfish interest in view, entirely forgetting the solemn obligation due their patrons for the confidence thus placed in them.

Gentlemen, this is no slander on my fellow men, the facts in the case will bear me out in this statement, humiliating as must be its mention, to every honorable man. And although we feel you would scorn to thus impose on those who place themselves in your hands, having confidence in your skill, and believing you would conscientiously perform the best operation in your power; we would warn you that there is no greater temptation than this in the path of the young man commencing his profession.

Hasty operations is the motto of too many in these times, but they buy their "ill gotten" riches at too dear a price.

Let me show unto you a more excellent way; honest operations secures true friends, and leaves a conscience "void of offence." Should the "evil spirit" ever thus tempt you, we would advise the posting of the "Golden Rule" over your dental case, so that on all such occasions you can read your duty, there so truly defined. Depend on it, gentlemen, in every occupation in life, the tree will be known by its fruit.

We would urge you to foster Associations for the promotion of Dental knowledge, where you will be benefited by the experience and opinions of others, and have your own theories tested by those capable of judging of their utility. The argument so often adduced against uniting in these Associations for mutual improvement is, want of time.

A good old Quaker once remarked to a young man who neglected duty for want of time. " Well friend, thee has all the time there is."

Gentlemen Graduates,—And now it affords me sincere gratification to present to you these Diplomas, conferring the degree of Doctor of Dental Surgery, to which by patient continuance in well doing you are so justly entitled. While they remind you that your studies here are at an end, they also admonish you that other and equally important duties begin. As you have acquitted yourselves with credit here, may you be equally successful in the battle of life, and finally finish your course with joy.

In connection with the Diplomas, as a parting gift from the Faculty, I also present to each of you a copy of the Holy Bible,—" Time's treasure and the wonder of the wise." It is the only book that recognizes the idea of the universal brotherhood of mankind; the only book that dispels the darkness of the " valley and shadow of death." By accepting its truths not as a creed, but reducing the same to practice, you can not fail to arrive at honor and distinction in life, and be known as Christian gentlemen.

